# Stereotactic body radiotherapy of lymph node metastases under MR-guidance: First clinical results and patient-reported outcomes

**DOI:** 10.1007/s00066-021-01834-w

**Published:** 2021-09-01

**Authors:** Fabian Weykamp, Charlotte Herder-Wagner, Sebastian Regnery, Philipp Hoegen, C. Katharina Renkamp, Jakob Liermann, Carolin Rippke, Stefan A. Koerber, Laila König, Carolin Buchele, Sebastian Klüter, Jürgen Debus, Juliane Hörner-Rieber

**Affiliations:** 1 Department of Radiation Oncology, Heidelberg University Hospital, Heidelberg, Germany; 2 Heidelberg Institute of Radiation Oncology (HIRO), Heidelberg University Hospital, Heidelberg, Germany; 3 National Center for Tumor diseases (NCT), Heidelberg University Hospital, Heidelberg, Germany; 4 Heidelberg Ion-Beam Therapy Center (HIT), Department of Radiation Oncology, Heidelberg University Hospital, Heidelberg, Germany; 5 Clinical Cooperation Unit Radiation Oncology, German Cancer Research Center (DKFZ), Heidelberg, Germany; 6 core center Heidelberg, German Cancer Consortium (DKTK), Heidelberg, Germany

**Keywords:** Pelvis, Patient acceptance, Abdomen, Organs at risk, Visability

## Abstract

**Objective:**

Stereotactic body radiotherapy (SBRT) is a noninvasive treatment option for lymph node metastases (LNM). Magnetic resonance (MR)-guidance offers superior tissue contrast and enables treatment of targets in close vicinity to radiosensitive organs at risk (OAR). However, literature on MR-guided SBRT of LNM is scarce with no report on outcome parameters.

**Materials and methods:**

We report a subgroup analysis of a prospective observational study comprising patients with LNM. Patients received MR-guided SBRT at our MRIdian Linac (ViewRay Inc., Mountain View, CA, USA) between January 2019 and February 2020. Local control (LC), progression-free survival (PFS) and overall survival (OS) analysis were performed using the Kaplan–Meier method with log rank test to test for significance (*p* < 0.05). Our patient-reported outcome questionnaire was utilized to evaluate patients’ perspective. The CTCAE (Common Terminology Criteria for Adverse Events) v. 5.0 was used to describe toxicity.

**Results:**

Twenty-nine patients (72.4% with prostate cancer; 51.7% with no distant metastases) received MR-guided SBRT for in total 39 LNM. Median dose was 27 Gy in three fractions, prescribed to the 80% isodose. At 1‑year, estimated LC, PFS and OS were 92.6, 67.4 and 100.0%. Compared to baseline, six patients (20.7%) developed new grade I toxicities (mainly fatigue). One grade II toxicity occurred (fatigue), with no adverse event grade ≥III. Overall treatment experience was rated particularly positive, while the technically required low room temperature still represents the greatest obstacle in the pursuit of the ideal patient acceptance.

**Conclusion:**

MR-guided SBRT of LNM was demonstrated to be a well-accepted treatment modality with excellent preliminary results. Future studies should evaluate the clinical superiority to conventional SBRT.

**Video online:**

The online version of this article contains one video. The article and the video are available online (10.1007/s00066-021-01834-w). The video can be found in the article back matter as “Supplementary Information”.

## Background and purpose

Stereotactic body radiotherapy (SBRT) enables the application of high tumoricidal irradiation doses while simultaneously sparing organs at risk (OAR). Through localized radiotherapy, systemic therapy might be postponed to improve quality of life [1]. Nonetheless, especially when treating lymph node metastases (LNM) in the abdomen or pelvis, surrounding intestines are put in jeopardy [2]. Especially lymph node metastases of prostate cancer are localized more frequently around or abut intestinal structures [3].

During the last few years, there has been a growing interest in SBRT of LNM, as high local control rates and favorable toxicity profiles have been reported [4]. However, standard image-guided SBRT via cone beam CT scan (CBCT) only offers limited soft tissue contrast [5]. Furthermore, counterintuitively, for SBRT of LNM, respiratory motion also has to be accounted for in some cases [6–9]. This can be conventionally achieved by using an internal target volume (ITV) concept, which nonetheless results in a larger target volume leading to a higher dose load within OAR [10]. Online MR-guided radiotherapy is a relatively new treatment solution and offers superior soft tissue contrast, with some systems being also able to provide gated dose delivery [11]. Thus, the target volume and OAR can be monitored live during beam-on time. Due to the relatively long treatment sessions with MR-guided radiotherapy, one should also be aware of patient acceptance [12].

Available data on MR-guided SBRT of LNM are scarce. Moreover, to the best of our knowledge, no clinical results are available on MR-guided radiotherapy of these patients, except for a single case report [13]. The purpose of our study was to provide first outcome data and report on patient acceptance of this novel treatment modality.

## Methods

The study presented here is a subgroup analysis of a prospective observational trial and comprises cancer patients with lymph node metastases treated with MR-guided SBRT using the MRIdian Linac (ViewRay Inc., Mountain View, CA, USA) between January 2019 and February 2020. SBRT was defined as a single fraction dose ≥4 Gy and a number of fractions ≤12, in accordance with the current guideline of the working group “Stereotactic Radiotherapy” of the German Society of Radiation Oncology [14].

### Treatment characteristics

A thorough description of our treatment simulation and treatment planning has been published previously [12]. Five patients in our presented study were also part of the respective publication. In short, treatment simulation at the MR-Linac started with the acquisition of three-dimensional (3D) MR images in either inspiration breath-hold or free breathing using the true fast imaging with steady-state precession sequence (TrueFISP), followed by planar cine-MRI in a sagittal plane to evaluate motion of the target structure. The acquired MR image data were used as the primary image set for treatment planning. The acquisition of image data at the MR-Linac also functioned as a first check for patients’ compliance.

Afterwards, a planning CT scan with contrast-enhanced and noncontrast-enhanced sequences was acquired in the planned treatment position. The gross tumor volume (GTV) was defined as the macroscopic tumor volume in the MRI scan and the coregistered CT scan. As proposed by the current German guidelines for patients with recurrent prostate cancer, all prostate cancer patients had undergone a prostate-specific membrane antigen positron emission tomography CT scan (PSMA-PET-CT) before treatment simulation using the MR-Linac [15]. Thus, GTV of all prostate cancer patients was additionally defined on the PSMA-PET-CT. Clinical target volume (CTV) equaled the GTV with additional 2 mm adapted to natural organ boundaries. Typical planning target volume (PTV) margins for CT-based SBRT of LNM are 5–7 mm [16, 17]. We chose a PTV margin of 3 mm, which has been established in this setting for MR-Linac treatment [18, 19].

Every day, image guidance was carried out via the onboard 3D MRI with identical settings (field of view, duration, pulse sequence, breathing instructions) as during the original MR simulation. Soft-tissue-based registration with the reference MR scan was applied and always registered directly on the GTV. For MR-gating in real time, the TrueFISP sequence was applied using one sagittal slice (four frames per second). If the lymph node metastasis was detectable on the TrueFISP sequence, the lesion was used directly as the gating structure (the region of interest [ROI]). In any other case, an anatomical surrogate structure surrounding the target lesion was defined as the gating structure. Then, a gating boundary was created by applying a ROI expansion of 3 mm. The irradiation process was interrupted if the target structure left the tolerance field around the gating boundary (set to 3%, if necessary up to 5% in exceptional cases). Patients were offered additional visual control during the gating process through an in-room monitor showing the live sagittal cine-MRI and could thus modulate their breathing [12]. For demonstration purposes, a video of this process can be found in the supplementary material section. In our described patient collective, no online treatment adaptation was performed, as it had not yet been implemented.

Prescribed irradiation doses were chosen depending on the size of the target volume as well as the proximity to organs at risk. If possible, three fractions of 9 Gy were used, prescribed to the conformally enclosing 80% isodose, covering at least 95% of the PTV. Target volumes in close proximity to radiosensitive structures (e.g. the small bowel) were irradiated with six fractions of 5 Gy prescribed to the conformally enclosing 80% isodose. One larger para-aortic metastasis was treated with five fractions of 10 Gy prescribed to the conformally enclosing 80% isodose, since there was no dose-limiting OAR in the proximity. Target coverage was reduced in case OAR dose constraints could not be met. Dose constraints were the following (for three fractions) and adopted from Hanna et al. [20]:

- Esophagus: 0.5 cc < 25.2 Gy
- Stomach/Duodenum: 0.5 cc < 22.2 Gy
- Small bowel: 0.5 cc < 25.2 Gy
- Sigma/Rectum: 0.5 cc < 28.2 Gy
- Kidney: Mean dose <8.5 Gy
- Spinal cord: 0.1 cc < 21.6 Gy
- Cauda equina: 0.1 cc < 24 Gy
- Central airways: 0.5 cc < 32 Gy
- Heart: 0.5 cc < 26 Gy
- Ureter: 0.5 cc < 40 Gy
- Bladder: 0.5 cc < 28.2 Gy
- Femoral heads: 10 cc < 21.9 Gy

A self-developed patient-reported outcome questionnaire (PRO-Q) was used to evaluate patients’ experience with the MR-Linac treatment (graded from 1–5, where 1 represents a completely positive and 5 a completely negative experience) [12]. In addition, our MR-Linac staff was surveyed about their judgement on each patient’s treatment performance (graded from 1–10, where 1 represents a facile and 10 a nearly inacceptable expenditure).

### Endpoints and statistical methods

Local control (LC), progression-free survival (PFS) and overall survival (OS) were calculated starting from the first day of the SBRT. LC was evaluated per lesion, whereas PFS and OS were calculated per patient. Toxicity was described using the Common Terminology Criteria for Adverse Events (CTCAE v. 5.0).

Following the study protocol, each patient was specifically assessed for presence of fatigue, nausea, vomiting, diarrhea, constipation, dyspnea, cough, skin disorder and pain. This evaluation took place before irradiation, at the last treatment day and at first follow-up. Patients with prostate cancer received a prostate-specific antigen (PSA) measurement 6–8 weeks after SBRT and then every 3 months. In case of a rising PSA level in two consecutive measures, patients were evaluated for receiving a new PSMA-PET scan as offered by current German guidelines to identify the exact location of the recurrence [15]. The other patients received a follow-up with a contrast enhanced MRI or CT scan, performed 6–8 weeks after the SBRT as well as a clinical examination. Further clinical and imaging follow-up was performed every 3 months at the discretion of the responsible oncologist and was not part of the prospective study.

LC, PFS and OS were estimated with the Kaplan–Meier method. Univariate analysis was performed with the log-rank method to test for significance. Median follow-up time was assessed using the reverse Kaplan–Meier method. The biologically effective dose (BED) was calculated applying the linear-quadratic model [21]. An α/β ratio of 3 was assumed for LNM of prostate origin, an α/β ratio of 10 was assumed for all other origins [3]. Statistical analysis was performed with SPSS software (Version 24.0, IBM, Armonk, NY, USA). A *p*-value of <0.05 was defined as significant. The MR-Linac observational study was approved by the ethics committee of the University Hospital (S-543/2018).

## Results

Median patient age was 70 years. Most patients treated had a very good Karnofsky performance status. The most common primary tumor was prostate cancer (72.4%), of which 66.7% had no further metastases and 33.3% had 1 to 3 further metastases, reflecting the lower metastastic burden in this patient group. Systemic therapy was present in 31.0% of the patients before and in 24.1% of the patients after SBRT. In prostate cancer patients, 28.6% had antihormonal therapy before and 28.6% after SBRT. Further patient characteristics are displayed in Table 1.

**Table 1:** Patient characteristics (*n* = 29)

**Median age**	70 years	Range 37–80 years
**Median Karnofsky score**	90%	Range 80–100%
**Median body mass index**	27.1 kg/m^2^	Range 21.2–35.2 kg/m^2^
**Female/male**	2/27	6.9%/93.1%
**Prostate cancer**	21	72.4%
**Other**	8	27.6%
*n* = 2 gastric cancer, *n* = 2 colorectal carcinoma, *n* = 1 adenoid cystic carcinoma, *n* = 1 urinary bladder carcinoma, *n* = 1 renal carcinoma, *n* = 1 tubal cancer		
**No distant metastases present**	15	51.7%
**Oligometastatic disease (*n*** **≤** **3 distant metastases)**	10	34.5%
**Oligoprogressive disease (*n*** **>** **3 distant metastases)**	4	13.8%
**Extranodal disease progression within 4 weeks before irradiation**	3	10.3%
*n* = 2 prostate cancer, *n* = 1 tubal cancer		
**Systemic therapy within 4 weeks before irradiation**	9	31.0%
*n* = 6 hormonal therapy, *n* = 3 chemotherapy		
**Systemic therapy within 4 weeks after irradiation**	7	24.1%
*n* = 6 hormonal therapy, *n* = 1 chemotherapy		
**Adverse events before radiotherapy**		
*Grade I*	12	41.4%
(Including: fatigue, pain, constipation, flatulence, nycturia, diarrhea, nausea, proctitis)		
*Grade II*	1	3.4%
(Proctitis)		
*Grade ≥III*	0	0
**Adverse events at last treatment day**		
*Grade I*	12	41.4%
(Including: fatigue, pain, constipation, flatulence, nycturia, diarrhea, nausea, cough)		
*Grade II*	1	3.4%
(Fatigue)		
*Grade ≥ III*	0	0
**Adverse events at first follow-up**		
*Grade I*	4	13.8%
(Including: fatigue, flatulence, dyspnea)		
*Grade II*	0	0
*Grade ≥ III*	0	0

**Fig. 1:**
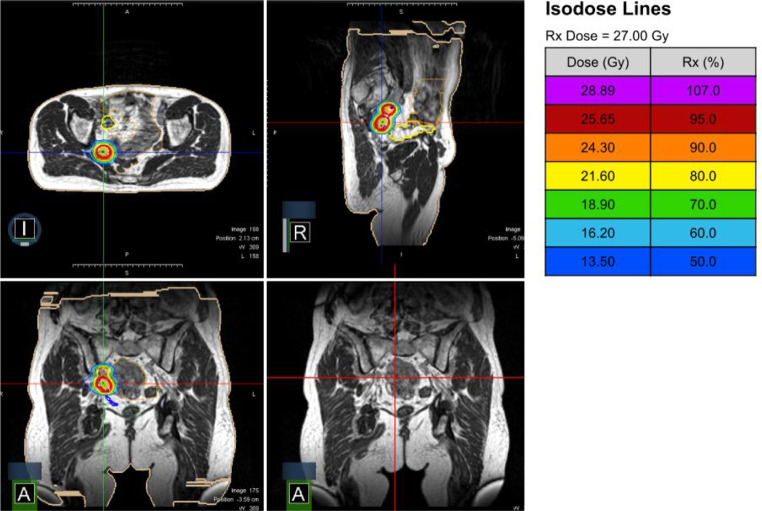
MR-Linac treatment plan (3 fractions of 9 Gy prescribed to the conformally enclosing 80% isodose) from different perspectives (*I* inferior, *A* anterior, *R* right) with and without isodose lines

Treatment characteristics are described in Table 2. Most patients received SBRT of a single lymphatic metastasis (69.0%), which was mainly located in the pelvis (67.7%). Median GTV and CTV size were 1.8 mL (0.1–70.8 mL) and 3.3 mL (0.4–81.7 mL) with a median prescription dose of 27 Gy (24–50 Gy). Fig. 1 shows a typical treatment plan of the pelvis. Moreover, it demonstrates the high soft-tissue contrast, enabling to distinct the treatment volume from the neobladder (yellow line) in the sagittal plane.

**Table 2:** Irradiation treatment characteristics

**Total number of irradiated lymphatic nodes per patient (*n*** **=** **39 lesions)**		
*n* *=* *1*	20	69.0%
*n* *=* *2*	8	27.6%
*n* *=* *3*	1	3.4%
**Localization of irradiated lymphatic nodes**	Mediastinal (14.7%), thoracic aorta (2.9%), retroperitoneal (14.7%), pelvis (67.7%)	
**Target volumes (*n*** **=** **34)**	**Median**	**Range**
*GTV*	1.8 mL	0.1–70.8 mL
*CTV*	3.3 mL	0.4–81.7 mL
*PTV*	10.0 mL	2.6–110.3 mL
**Prescribed total dose**	27 Gy	24–50 Gy
**Fractions**	3	3–6
**GTV D50**	33.0 Gy	23.6–51.6 Gy
**EQD2 and BED for prostate histology (*n*** **=** **26)**		
*EQD2 (α/β* *=* *3)*	64.8 Gy	48.0–64.8 Gy
*BED (α/β* *=* *3)*	108.0 Gy	80.0–108.0 Gy
**EQD2 and BED for other histology (*n*** **=** **8)**		
*EQD2 (α/β* *=* *10)*	37.5 Gy	37.5–130.0 Gy
*BED (α/β* *=* *10)*	45.0 Gy	45.0–216.7 Gy
**Treatment time (“on table”)**	43.0 min	26.0–93.0 min
*Radiation time*	11.0 min	7.0–26.0 min
*Beam on time per fraction*	3.5 min	1.7–5.0 min

*BED* biologically effective dose, *CTV* clinical target volume, *EQD2* equivalent dose at 2 Gy, *GTV* gross tumor volume, *PTV* planning target volume

### Outcome

**Fig. 2:**
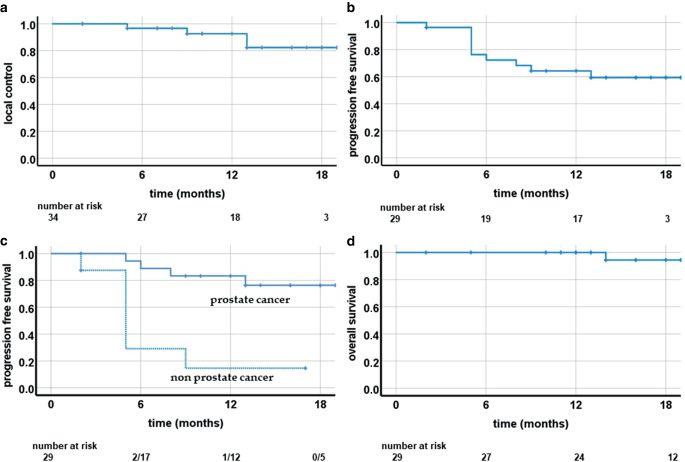
Local control (**a**), progression-free survival (**b**) divided by prostate cancer histology (**c**) and overall survival (**d**) following magnetic resonance (MR)-guided stereotactic body radiotherapy (SBRT) of lymph node metastases

Median follow-up was 13.0 months. Estimated LC was 92.6% at 12 months (Fig. 2a). Three patients suffered from local recurrence (*n* = 1 prostate cancer, *n* = 1 colon carcinoma, *n* = 1 urinary bladder cancer). In these three cases, the irradiated LNM itself had recurred. One additional patient had nodal recurrence (prostate cancer), at distance from the irradiated LNM. PFS at 12 months was 67.4% (Fig. 2b) and was higher in prostate cancer patients than in nonprostate cancer patients (83.3% vs. 14.6%, *p* < 0.01; Fig. 2c). One patient died during follow-up. Estimated OS at 12 months was 100.0% (Fig. 2d).

### Toxicity

For detailed toxicity data, please see Table 1. Nearly half of the patients already had mild complaints before starting SBRT (43.4%; CTC grade I–II). Compared to baseline, 6 patients (20.7%) developed new grade I toxicities on the last day of radiotherapy (fatigue, pain, constipation, flatulence, nycturia, diarrhea, nausea and/or coughing). One grade II toxicity occurred (fatigue). No adverse event grade III or higher was reported at any time.

### Patient- and staff-reported outcome

Table 3 shows patient- and staff-reported outcome. Treatment at the MR-Linac was accepted particularly well, especially overall treatment experience and items regarding the staff were rated very positively (each median 1 point, no rating >2). The low room temperature was not well tolerated (median 3 points). The median time to physical and mental recovery after the first treatment session were 0 min (range 0–300 min). The MR-Linac staff reported rather low treatment expenditure (median 3 points).

**Table 3:** Patient- (items 1–18) and staff (item 19)-reported outcome (available for *n* = 23 patients)

	Median	Range
*Categorical point scale form 1–5, where 1 equals very positive and 5 equals very negative*		
1. Overall treatment experience	1	1–2
2. Information provided by the staff	1	1–2
3. Friendliness of the staff	1	1
4. Duration of the treatment	2	1–4
5. Size of the MRI bore	2	1–4
6. Positioning during radiotherapy	2	1–5
7. Having to lie still	2	1–3
8. Noise in the MR-Linac	2	1–4
9. Temperature in the MR-Linac	3	1–4
10. Local temperature of body parts	3	1–4
11. Tingling sensations in fingers and toes	1	1–5
12. Breathing instructions	1	1–3
13. Breath holding	1	1–3
14. Anxiousness during treatment session	1	1–3
15. Reported time until full recovery after the radiotherapy session	0 min	0–300 min
16. Difficulty to hold the target with one’s own breath	1	1
17. Ability to watch one’s own treatment via monitor	1	1
18. Feeling of having active control over the treatment duration	1	1–2
*Categorical point scale from 1–10, where 1 equals very positive and 10 equals very negative*		
19. Treatment complexity from the perspective of the staff	3	1–9

## Discussion

The presented subgroup analysis of a prospective observational study comprises 29 patients with a total of 39 lymph node metastases treated with ablative MR-guided SBRT from January 2019 to February 2020. Most patients were diagnosed with prostate cancer and showed a very good Karnofsky performance status, with a median age of 70 years.

Irradiating lymph node metastases via online MR-guidance has been proven to be feasible [22]. However, data are only available from one other study group evaluating MR-guided SBRT using the 1.5 T Elekta MR-Linac: Winkel et al. retrospectively compared high field (1.5 T) MR-guided irradiation of lymph node metastases with conventional CBCT-Linac treatment in 20 patients and demonstrated fewer unplanned violations of OAR constraints [19, 22]. The same study group also successfully evaluated the utilization of a vacuum cushion for MR-guided pelvic/para-aortic lymph node SBRT to reduce intrafractional motion [23]. However, to the best of our knowledge, there has not yet been any study about clinical results and patient-reported outcome following MR-guided SBRT of lymph node metastases.

By contrast, conventional SBRT of lymph node metastases has been shown to be effective and safe in prostate cancer, with a LC rate at 24 months of 84% and no grade III toxicity or higher [24]. Another study including patients with prostate cancer LNM described 98% LC during a median follow-up of 30 months, with only one case of grade III toxicity [25]. CBCT-SBRT of LNM was also proven to be effective for various primary tumors, with LC rates after 1 year of up to 98% and a comparably favorable side effect profile [16, 17]. Table 4 summarizes the larger studies on radiotherapy of lymphatic node metastases with CBCT- or MR-Linac-guided SBRT. With a median of 27 Gy in three fractions (median BED 51.3 Gy), our dose prescription appears to be rather lower. However, we performed a prescription to the conformally enclosing 80% isodose, covering at least 95% of the PTV, to allow for a steeper dose gradient. This leads to a higher dose in the GTV and a dose maximum of 125%. Most studies in the field use a prescription to the 98% or 95% isodose, which does not include a dose escalation in the GTV and impairs a direct comparison of the simple prescription doses.

**Table 4:** Larger studies on stereotactic body radiotherapy of lymphatic node metastases including MR-guided radiotherapy

	Patients, characteristics, design	Radiation technique	Toxicity	LC	PFS	OS
**Jereczek-Fossa et al. (2017) **[24]	Patients: *n* = 94 (100% prostate cancer) with ≤4 oligometastases treated lymph node metastases: *n* = 124 (70% single lesion; 39.5% extrapelvic) median follow-up 18.5 months median age: 70 y KPS 100 = 70% 100% male retrospective design	Median dose of 24 Gy in 3 fractions concomitant androgen depriving therapy: 36% GTV = CTV PTV margin: 2 mm (Cyberknife) and 3 mm (Vero system SBRT) median CTV volume: NA median dose: NA	14% grade I–II 0% ≥ grade III	84% @ 2 years	30% @ 2 years	NA
**Franzese et al. (2020a) **[16]	Patients: *n* = 278 (20.9% colorectal, 18.0% prostate cancer) with ≤5 oligometastases (25% had extranodal disease) treated lymph node metastases: *n* = 418 (76.7% single lesion) median follow-up of 15.1 months median age: 67 y (31–89) ECOG 0: 68.3% 63% male retrospective design	Volumetric modulated arc therapy mostly 45 Gy in 6 fractions with a median BED10 of 78.75 Gy systemic therapy before SBRT: 54.4% GTV = CTV PTV margin 5–7 mm median CTV volume 8.55 mL (range 0.1–150.9) median dose (BED10Gy): 78.7 Gy (37.5–105.6)	16% grade I 2.2% grade II 0.4% grade III (gastrointestinal) 0% ≥IV°	87.2% @ 1 year 76.8% @ 2 years	NA	NA
**Franzese et al. (2020b) **[17]	Patients: *n* = 52 (75% genitourinary cancer, 21% gastrointestinal) with 1 to 3 lymph node metastases (11.5% had extranodal disease) treated lymph node metastases: *n* = 64 (81%% single lesion) median follow-up: 24.4 months median age 70 y (50–88) ECOG 0: 73% 88.5% male prospective phase II trial	Volumetric modulated arc therapy 48 Gy in 4 fractions for all lesions systemic therapy before SBRT: 48% GTV = CTV PTV margin 5 mm median CTV volume 2.21 mL (0.14–61.3) median dose (BED10Gy): 105.6 Gy	7.7% grade I (pain, fatigue, nocturia and dysuria) 0% grade ≥ II	97.9% @ 1 year 82.1% @ 2 years	67.4% @ 1 year 42.4% @ 2 years	97.3% @ 1 year 94.2% @ 2 years
**Winkel et al. (2020) **[19]	Patients: *n* = 20 target coverage and dose criteria-based evaluation	1.5 T MR-Linac (Unity, Elekta AB, Stockholm, Sweden) utilization of online adaptation 5 × 7 Gy for all lesions GTV-PTV margin: 3 mm	NA	NA	NA	NA
**Werenstijn-Honingh et al. (2020) **[18]	Patients: *n* = 39 evaluation of a vacuum cushion to reduce intrafractional movement	1.5 T MR-Linac (Unity, Elekta AB, Stockholm, Sweden) utilization of online adaptation 5 × 7 Gy for all lesions GTV-PTV margin: 3 mm	NA	NA	NA	NA
**Weykamp et al. (present study)**	Patients *n* = 29 (*n* = 21 prostate cancer, *n* = 2 gastric cancer, *n* = 2 colorectal carcinoma, *n* = 1 adenoid cystic carcinoma, *n* = 1 urinary bladder carcinoma, *n* = 1 renal carcinoma, *n* = 1 tubal cancer) (27% had extranodal disease) treated lymph node metastases: *n* = 39 (69% single lesion; 32.3% extrapelvic) median age: 70 y (37–80 y) KPS 100 = 41% female: 6.9% median follow-up: 13.0 m (2–22) subgroup analysis from a prospective observational study	MRIdian Linac (ViewRay Inc., Mountain View, CA) 0.35 T step-and-shoot IMRT; utilization of gating median dose 27 Gy (range 24–50 Gy) in 3 fractions (range 3–6) prescribed to the enclosing 80% isodose, covering at least 95% of the PTV volume concomitant androgen depriving therapy (in case of prostate cancer): 28.6% CTV = GTV + 2 mm PTV = CTV + 3 mm adaptive technique: no median of radiation time: 11.0 min (7.0–26.0 min) median treatment time 43.0 min (26.0–93.0 min) median CTV volume 3.3 mL (0.3–81.7 mL) median dose (BED10Gy): 51.3 Gy (43.2–100.0 Gy)	New compared to baseline: 20.7% grade I (fatigue, pain, constipation, flatulence, nycturia, diarrhea, nausea, cough) 3.4% grade II (fatigue) 0% grade ≥ III	92.6% @ 1 y	64.3% @ 1 y	100.0% @ 1 y

*BED* biologically effective dose, *CTV* clinical target volume, *EQD2* equivalent dose at 2 Gy, *GTV* gross tumor volume, *m* months, *min* minute, *mm* millimeter, *MRI* magnetic resonance imaging, *NA* not available, *PTV* planning target volume, *y* years

Treatment toxicity of our cohort was comparable to CBCT-guided SBRT with only one case of grade II toxicity (fatigue) and no grade III toxicity or higher [16, 17, 24]. Local control of the irradiated lymph nodes was high in our study group with a rate of 92.6% after 12 months, which lies within the range of the aforementioned studies (87.2–97.9%). PFS was 64.3% at 12 months. As previously described, prostate cancer patients had a superior PFS to nonprostate cancer patients (83.3% vs. 14.6%, *p* < 0.01; Fig. 2c; [17, 26]). In a systematic review of 211 prostate cancer patients receiving SBRT for LNM, antihormonal therapy was present in 40.5% of the patients vs. 28.6% in the presented study. SBRT might offer the possibility to postpone systemic therapy and hence improve quality of life [1].

Although, clinical results of conventional SBRT for LNM are satisfying, further treatment optimization is warranted [2]. MR-guided radiotherapy is thought to become a potential practice changing technology in the treatment of various tumor entities, as it offers superior soft tissue contrast for the precise identification of the target volume and detection of inter- and intrafractional changes in adjacent OAR. This new versatile technology thus supports the delivery of high irradiation doses while sparing OAR [27–29]. Gated dose delivery allows for tighter OAR margins and further reduces the proportion of irradiated healthy tissue [10]. About two third (67.7%) of the irradiated lymph nodes metastases in our study cohort were localized in the pelvis. Nonetheless, we performed gated dose delivery also in these patients to gain insight into breathing motion, which then played a minor role in daily practice.

Despite a rather long treatment time (median 43.0 min) lying on a nonpadded treatment couch, patients reported an excellent overall treatment experience, with no rating being below the second-best possible grade (Table 3). MR-guided radiotherapy is staff and time intensive [27], while beam on time (median 3.5 min) represents only a small proportion of the overall treatment time (median 43.0 min). For technical reasons, temperature is cooled down in the treatment room. Although patients who tend to feel cold easily are allowed to wear nonmetal personal clothes under the standardly worn medical scrubs or are provided with an additional blanket, the low temperature is still the greatest obstacle concerning the pursuit of the ideal subjective treatment experience (median grade 3). Given the described hindering circumstances of long treatment duration, small MRI bore and low room temperature, the patient reported experience with the gating process was rated surprisingly positive (median grade 1, range 1–3). Most patients needed no recovery time after the respective daily treatment sessions. Having the tumor displayed in front on oneself as part of the gating process did in general not cause anxiety in the patients, yet even seemed to provide a feeling of power and control. One patient described this setting as highly satisfying and relieving, being able to act directly and actively against the tumor after several passive months of fearing his tumor to recur.

A limitation of the presented study is its rather small sample size and its, at the present time, short follow-up. Nonetheless, our presented patient cohort is the first study in the field, to the best of our knowledge. Moreover, toxicity data were gathered prospectively, while long-term experience is still immature. Another limitation of this study is the utilization of an in-house designed, not externally validated questionnaire. Our questionnaire was specifically designed for our institution. Next to evaluating patients’ satisfaction and feasibility of MR-Linac treatment, we used the tool as a quality assessment instrument to measure our staff’s performance and opinion. Since there was no control group, patients could have had a high level of satisfaction in the first place. Undeniably, there is an underlying selection bias, with patients being excluded from the study due to claustrophobia or pacemaker devices, which might have influence on the patient-reported outcome.

Online adaptive MR-guided SBRT, a procedure where a new treatment plan is created before each irradiation session based on daily anatomy changes, is to date described for liver, adrenal, pancreatic and lung tumors as well as lymph node metastases [18, 30–37]. Online adaption was implemented into our clinical workflow in February 2020 and has since been used daily for every patient, including patients with lymph node metastases. First experience with high field (1.5 T) MR-guided pelvic/para-aortic lymph node SBRT is promising [19]. The PTV coverage was herein shown to be higher with an adaption to the shape of the daily anatomy rather than with an adaption to the mere patients’ position [38]. Nonetheless, it needs to be kept in mind that adaption will further prolong the already demanding treatment procedure.

However, also without daily adaption, MR-guided SBRT enables the visualization of the target volume itself as well as the surrounding OAR. Hence, also LNM close to critical OAR can be visually separated through the high soft-tissue contrast of the MRI and treated safely (Fig. 1).

## Conclusion

MR-guided SBRT of lymph node metastases represents a particularly well-accepted treatment modality as measured by our patients’ questionnaire. Local control was excellent with only mild toxicity. Our results confirm the need for prospective studies to identify patients, in which OAR would otherwise have hindered SBRT.

## Supplementary Information

**Supplement Video **Gated irradiation of a pelvic lymphatic node metastasis at the MR-Linac (*green* gating structure; *red* gating boundary). The patient was instructed to breath in, then out and then to hold the breath for as long as possible
